# Amplitude-Integrated EEG for Neurological Assessment and Seizure Detection in a German Pediatric Intensive Care Unit

**DOI:** 10.3389/fped.2019.00358

**Published:** 2019-08-28

**Authors:** Nora Bruns, Iciar Sanchez-Albisua, Christel Weiß, Eva Tschiedel, Christian Dohna-Schwake, Ursula Felderhoff-Müser, Hanna Müller

**Affiliations:** ^1^Department of Pediatrics I, Neonatology, Pediatric Intensive Care, Pediatric Neurology, University Hospital Essen, University Duisburg-Essen, Essen, Germany; ^2^Department of Medical Statistics and Biomathematics, University Hospital Mannheim, University of Heidelberg, Mannheim, Germany; ^3^Division of Neonatology and Pediatric Intensive Care, Department of Pediatrics, University Hospital Erlangen, University of Erlangen-Nuremberg, Erlangen, Germany

**Keywords:** amplitude-integrated EEG, pediatric critical care, neuromonitoring, aEEG, outcome, seizure, continuous EEG

## Abstract

**Objective:** The aim of our study was to assess the use of aEEG in our pediatric intensive care unit (PICU), indications for neuromonitoring and its findings, utility for seizure detection, and associations with outcome.

**Design:** We retrospectively analyzed non-neonates who were treated in our PICU and received amplitude-integrated EEG (aEEG).

**Patients:** 27 patients aged between 29 days and 10 0/12 years (median 7.3 months) were included, who received a total of 35 aEEGS.

**Measurements:** aEEG tracings were assessed for background (BG) pattern and its evolution, seizures, and side differences using a visual classification (Hellström-Westas). Clinical data were collected from patients' histories and analyzed for correlation with aEEG findings.

**Main results:** While rare in early years, there was an increase in use over time. Most aEEGs were conducted because of (suspected) seizures or for management of antiepileptic treatment. aEEG had low sensitivity but high specificity for recognition of pathological BG pattern with reference to conventional EEG. Worsening of BG pattern or failure to improve was associated with death. Seizure detection rates by aEEG were higher than by clinical observation, especially for identification of non-convulsive epileptic state (ES). Side differences in aEEG were rare, but if present, they were associated with unilateral brain injury.

**Conclusions:** aEEG is useful for the detection of seizures and ES in pediatric intensive care patients. Abnormal BG pattern and poor evolution of BG are negatively associated with survival. aEEG is a potential supplement to conventional EEG, facilitating long-term surveillance of cerebral function when continuous full-channel EEG is not available. Further investigation is needed.

## Introduction

A common problem in pediatric intensive care is the neurological assessment of children with altered mental state. Additionally, patients often require sedation or opiate analgesia, making early recognition of neurological complications prompting immediate treatment even more difficult. Several scoring systems to assess vigilance, pain, distress, and withdrawal have been established, but the evaluation of vigilance remains a challenge. Additionally, transport of critically ill patients for cranial imaging collides with the idea of minimal handling and may not be available or medically possible at any time.

Apparative methods for indirect brain monitoring in the pediatric intensive care unit (PICU) include measurement of cerebral tissue oxygenation, cerebral blood flow, and cerebral metabolic state. Monitoring of electrical activity is possible by bispectral index (BIS-) monitoring and conventional continuous electroencephalography (cEEG) (1, 2). However, each technique has its limitations regarding duration, accuracy, availability, or easy applicability. cEEG is considered the best approach to monitor electrocortical activity in pediatric patients, but remains a scarce resource as it requires interpretation by an epileptologist. An alternative device that is broadly available in neonatal intensive care units (NICUs) is a simplified and time-compressed electroencephalogram—amplitude-integrated EEG (aEEG). It was initially developed for neuromonitoring in adult intensive care and nowadays has become a routine method in neonatal intensive care (3–7). The precedent from neonatology shows that it can be interpreted by non-epileptologists and is useful to direct care and predict outcome (4, 8–11). At the same time, aEEG is becoming more and more popular for prediction of outcome after cardiac arrest in adult intensive care (12–15). Despite its broad application in neonatal patients, very little has been reported about the use of aEEG in general pediatric intensive care. On the other hand, literature supporting the use of continuous EEG in pediatric critical care after cardiac arrest, in patients with altered mental status, and for the detection of non-convulsive seizures increased considerably during recent years (16–22). Time to cEEG and presence of non-convulsive epileptic state have been found to be independent factors associated with mortality in neonates and pediatric critical care patients (18). Given the limited availability of continuous full-channel EEG monitoring, it may be worth looking for alternative methods for bed-side assessment of electrocortical activity and seizures in pediatric critical care patients.

The aim of our study was to assess the evolution of aEEG use in our PICU over the past 9 years, the findings in aEEG compared with conventional EEG, the utility of aEEG for seizure detection, therapeutic consequences drawn from the recordings, and possible associations with adverse outcome.

## Methods

### Patient Recruitment

All children treated in the PICU of the University Hospital Essen, Germany, who received an aEEG in our PICU between 07/2009 and 07/2018 were eligible for our retrospective analysis. Neonates and preterm infants before 44 weeks' corrected gestational age were excluded. The study was approved by the ethics committee of the Medical Faculty of the University of Duisburg-Essen (Ethik-Komission der Medizinischen Fakultät der Universität Duisburg-Essen).

### aEEG Recording

As our PICU does not have a standardized protocol for the application of aEEG, the indication was set by the physician in charge. All aEEGs were recorded by the nurse or the doctor in charge of the child. The aEEG was recorded as a two channel EEG using mainly needle electrodes, and in some cases hydrogel electrodes or gold caps with BRM2 and BRM3 monitors (BrainZ Instruments, Auckland, Auckland Council, New Zealand) and Braintrend monitors (MT Monitortechnik, Hannover, Niedersachsen, Germany). Electrodes were placed on the scalp corresponding to the positions C3, P3, C4, and P4 of the international 10–20 system. A reference electrode was placed on the patients' back or forehead (23).

### aEEG Interpretation

All aEEGs were interpreted by two independent investigators (N.B. and H.M.) blinded to the clinical course and outcome. The tracing was divided into 4 h sections, of which each section was checked for quality (no relevant artifacts, impedance <15 kΩ). Each investigator analyzed the background pattern, seizures, and side differences using the classification by Hellström-Westas independently (24). In case of disagreement, the section in doubt was reassessed by the two raters together and consent was sought. Interrater agreement was not assessed separately, but we previously published good agreement between the two raters for the classification of Hellström-Westas [weighted κ = 0.7589 (CI: 0.7177–0.8001)] (8).

#### Background Patterns

Background patterns were distinguished into continuous normal voltage (CNV), discontinuous normal voltage (DC), continuous low voltage (CLV), burst suppression (BS), and flat trace (FT) according to the original publication (24). CNV was considered as a normal background pattern, whereas all other background patterns were considered abnormal. The predominant background pattern was used for statistical analysis.

#### The Evolution of Background Pattern

The evolution of background pattern was assessed and categorized according to the suggestion by Sewell et al. which was developed for evaluation after perinatal asphyxia (25). For this purpose, we compared the very first and the very last section that was available for each infant. When several tracings were performed, we used the first section of the first tracing and last section of the last tracing. The evolution was classified as follows: Persistently normal (CNV at the beginning and end of tracing); normalization (not continuous at beginning but at the end of tracing); persistent mild abnormality (CNV/DC at start and DC at end of tracing); progression to severe [CNV/DC at start, but progressed to abnormal background (CLV, BS, FT) at end of tracing]; improved without normalization [abnormal pattern (CLV, BS, FT) at start that improved to DC]; persistently severe [abnormal background pattern (CLV, BS, FT) at start and end of tracing]; too short (only one section available).

#### Electrographic Seizures

Electrographic seizures were identified by an abrupt rise in the minimum amplitude (and mostly also the maximum amplitude). They were confirmed by examining the raw EEG for simultaneous seizure activity for at least 10 seconds (sharp waves, spikes or gradual build-up and following decline in frequency and amplitude). According to the frequency of appearance they were classified as single seizure, repetitive seizures (3 or more seizures within the tracing), and epileptic state (ES) (continuous or highly repetitive seizure activity for more than 30 min). The highest seizure classification was used for statistical analysis.

#### Side Differences

Side differences were documented for each tracing.

### Clinical Data

Clinical data were extracted retrospectively from medical records. Clinical seizures were defined as focal or generalized tonic, clonic, or tonic-clonic muscle activity for at least 10 seconds and in >90% confirmed by a second observer. Clinical seizures were documented in the patient's history and classified as single seizure, repetitive seizures (3 or more clinical seizures during the aEEG recording), and epileptic state (continuous or highly repetitive seizure activity for at least 30 min).

### Statistical Analysis

Fisher's Exact Test was performed to assess the association of the binary outcome survival (yes or no) with the following parameters: gender, neurologic symptoms at admission, sedation, mechanical ventilation, cranial imaging (CT/MRI scan), electrographic seizures, clinical seizures, pathological background pattern, pathological EEG, side difference in at least one aEEG section with a unilateral process shown in imaging, and evolution of background pattern. Kappa coefficient as a measure of agreement has been assessed in order to compare clinical with electrographic seizures. All statistical calculations have been performed using SAS software, release 9.4 (SAS Institute Inc., Cary, NC, USA) and the result of a statistical test has been considered as significant for *p* < 0.05.

## Results

**Figure 1:**
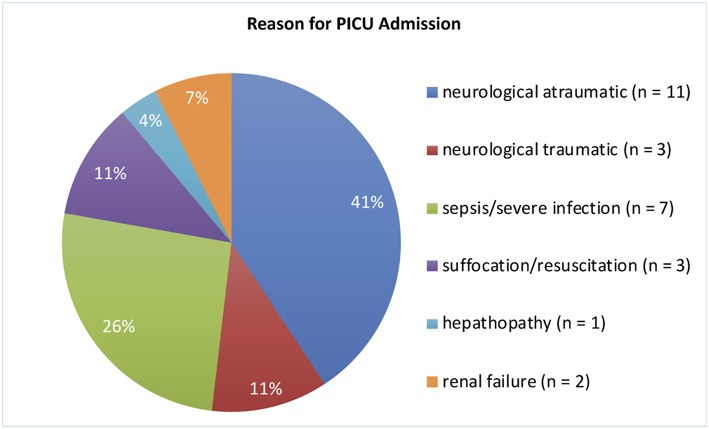
Reasons for PICU Admission. The most severe diagnosis was selected if more than one was applicable.

We included 29 children, of which 2 had to be excluded due to bad quality of aEEG recordings. The remaining 27 children (16 [59%] male) aged between 29 days and 10 0/12 years at aEEG recording (median: 7.3 months). Weight upon admission was 3.6–36.0 kg (median 6.8 kg). Neurological symptoms were the reason for hospital admission in 15 infants (56%). Cerebral imaging (MRI/CT) were performed in 18 (51%) children, confirming unilateral intracranial lesions in 5 (14%) patients. 1 (3%) infant received cerebral ultrasound only. None of our patients was admitted to the PICU because of underlying cardiac symptoms/disease or underwent cardiac surgery during the stay (Figure 1 and Table 1). 22 (81%) patients survived. An overview regarding reasons for PICU admission and the patients' clinical details are provided in Figure 1 and Table 1. If several categories of PICU admission were applicable, the most severe diagnosis has been selected (Figure 1). Five patients (19%) died. The reasons for death were brain death with consecutive withdrawal of care (*n* = 1), palliative care in progredient neurodegenerative disease (*n* = 3), and *mors in tabula* during craniectomy in imminent brain death (*n* = 1).

**Table 1:** Clinical data of patients.

**No**.	**Sex, age, weight, age at death**	**Acute condition**	**Pre-existing conditions and complications**	**Neurological complications and neurosurgical interventions**	**Indication for aEEG**	**aEEG background at beginning/end**	**Seizures in aEEG**
1	♂, 1 month, 3.6 kg; ^*†*^2 months	Apparent life-threatening event, resuscitation	Undiagnosed Leigh's syndrome	Hypoxic brain injury, 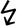	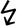	DC-C	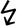
2	♀, 12 months, 10.5 kg	Epilepsy	Hearing loss, bronchiolitis (RSV)	None	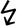 , sudden coma	C-C	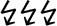
3	♂, 7 months, 7.2 kg	Intraventricular hemorrhage	Vein of galen malformation (partial embolization), intracranial hemorrhages, epilepsy, stenosis of aortic valve, and trachea, mechanical ventilation at night	Brain edema—EVD	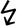	C-C C-C	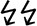 -
4	♂, 12 months, 10.0 kg	Popcorn aspiration, resuscitation	None	Hypoxic brain injury, 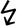	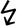	C-C	-
5	♀, 9 months, 8.1 kg; ^*†*^9 months	Hemolytic uremic syndrome (HUS)	Acute renal failure, dialysis, dehydration, electrolyte imbalances, hematochezia	Brain edema—EVD	Neuromonitoring	C	-
6	♂, 3 months, 5.0 kg	Acute renal failure	Rhabdomyolysis	None	Reduced vigilance	C-C	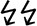
7	♂, 8 months, 11.0 kg	Pneumonia, sepsis	West syndrome, iatrogenic cushing syndrome	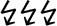	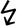	BS-BS	-
						C-CLV	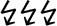
8	♀, 4 months, 5.7 kg	Left-sided intracranial hemorrhage	Progressive familiary intrahepatic cholestasis, vitamin K deficiency	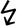 —trepanation and hematoma removal	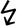 , anisocoria	Left: FT-FT Right: C-C	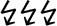 -
9	♂, 1 month, 4.2 kg	Seizures	Complex heart defect	None	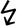	C	-
10	♀, 10 months, 8.0 kg; ^*†*^ 10 months	Febrile seizure, resuscitation, metabolic encephalopathy, RSV bronchiolitis	None	Brain edema, 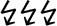 —bifrontal craniotomy	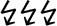	BS-FT	-
11	♀, 4 months, 3.9 kg; ^*†*^ 5 months	RSV bronchiolitis, respiratory insufficiency	Mitochondriopathia, dystrophy, multiorgan failure	None	Neuromonitoring during severe illness	FT-DC	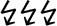
12	♂, 2 months, 4.7 kg	RSV bronchiolitis, respiratory insufficiency	Congenital stiff man syndrome, bone deformities, subdural hematoma, sepsis	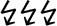	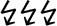	C-FT	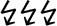
13	♂, 24 months, 11.0 kg	Resuscitation	Former preterm infant 34 + 6, esophageal atresia, dystrophy, metabolic derailment, dialysis	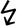 , multiple intracranial bleedings, hypoxic brain injury	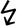 , neuro-monitoring after resuscitation	C-BS	-
14	♂, 5 months, 6.1 kg	Hygroma	Battered child syndrome, retinal bleedings	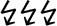 , acute and chronic hematoma, cerebellar infarction—EVD	Preceded 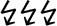 , neurosurgical intervention	CLV-CLV	-
15	♀, 3 months, 6.4 kg	Seizures with apneas	Resolved hyperbilirubinemia and kernicterus	None	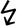 with apneas	C-C C	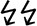 -
16	♀, 15 months, 5.7 kg	Hygroma	Suspected glutaraciduria	Brain atrophy, subdural hygroma—EVD	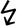	C	-
17	♂, 5 months, 6.8 kg	Epiletic state	Battered child syndrome, retinal and vitreous bleedings, multiple fractures of skull, ribs, and tubular bones	Multiple intracranial bleedings and ischemias	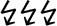	C-C C-C C-C	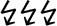 - -
18	♂, 5 months, 5.5 kg	Liver transplant	Biliary atresia, liver cirrhosis, severe urinary retention, dystrophy, intraoperative thrombosis, resuscitation, primary non-function of transplant, mass transfusion, re-transplantation, dialysis, chylascites, sepsis	First liver transplant: large infarction, intracranial bleedings, re-transplantation: intracranial bleedings, hydrocephalus—after re-transplantation: Ommaya reservoir, EVD, ventriculoperitoneal shunt	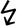	C-C C	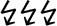 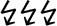
19	♀, 11 months, 7.0 kg; ^*†*^12 months	Respiratory insufficiency, arterial hypertonia	Unknown progressive neurodegenerative disease	None	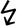	DC-C	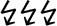
20	♂, 7 years and 11 months, 36.0 kg	Epileptic state, febrile infection-related epilepsy syndrome	Respiratory insuffiency, arterial hypotonia	None	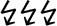	C-DC	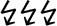
21	♀, 10 years and 1 month, 31.0 kg	Epileptic state	Structural epilepsy of the left hemisphere after congenital ventricular cyst	None	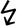	DC-DC	-
22	♂, 2 months, 5.3 kg	Intoxication with lorazepam	Neonatal epileptic encephalopathy (SCN2A mutation)	Coma	Coma, surveillance of response to antidot	BS-DC C-C	- -
23	♂, 4 months, 8.6 kg	Subdural hematoma	Suspected battered child syndrome, hematoma, retinal bleedings	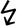	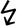	DC-C	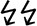
24	♂, 11 months, 5.5 kg	Pneumonia	Former preterm infant, chromosome aberration, agenesis of corpus callosum, hydrocephalus, neonatal seizures, pulmonary hypertension, cardiorespiratory failure	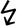	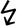	C-C transient left DC	-
25	♀, 1 month, 4.4 kg	Seizures	GLUT1 deficiency syndrome	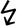	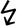	C-C	-
26	♀, 2 years and 9 months, 10.0 kg	Drowning, resuscitation	Pneumonia	Brain edema, 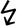 —bifrontal craniotomy	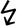	C-C	-
27	♂, 19 months, 12.0 kg	Aspiration pneumonia	Herpes encephalitis	None	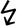	C-C	-

*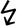, single seizure; 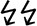, repetitive seizures; 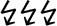, epileptic state*.

† *, deceased; ♀, female; ♂, male*.

### Conduction of aEEG

**Figure 2:**
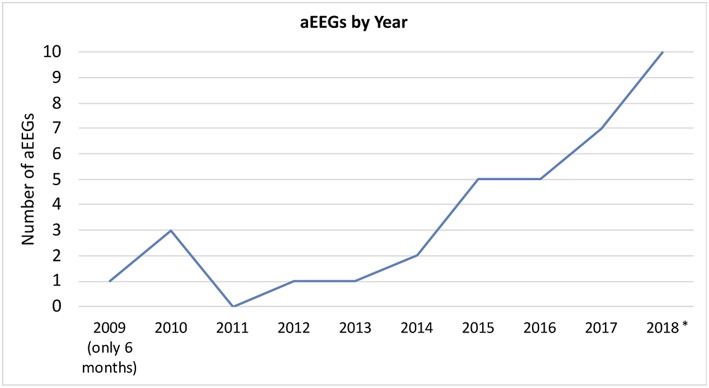
aEEGs by year. *In 2018, two patients received multiple nearly continuous aEEG recording for approximately weeks/months. These files were only counted as one recording per patient. aEEGs from 08/2018 to 12/2018 were counted, even though they were not analyzed for the study.

A total of 35 aEEGs was recorded with a range of duration from 4 to 96 h (median: 20 h) of evaluable tracing (please see Figure 2 for an overview over the conducted aEEGs per year). The indication for aEEG recording was suspected seizures or treatment guidance in 28 aEEGs (80%), reduced vigilance in 4 (11%), and others in 3 (9%).

17 (49%) aEEGs were recorded under mechanical ventilation, 6 (17%) under non-invasive respiratory support, and 12 (34%) under spontaneous breathing. Sedation was applied during 22 recordings (63%). Neurosurgical intervention was performed in 8 (30%) children. Performed surgeries included bifrontal craniotomy (*n* = 2), extraventricular drainage (EVD) (*n* = 4), removal of intracranial hematoma (*n* = 1), and in one patient the combination of Ommaya reservoir, EVD, and a ventriculoperitoneal shunt. Six surgeries were performed before aEEG recording, and 2 were performed after aEEG recording. In the child that underwent three surgeries, one aEEG was recorded directly after implantation of the Ommaya reservoir.

### aEEG Findings

#### Background Pattern and Comparison to EEG

**Figure 3:**
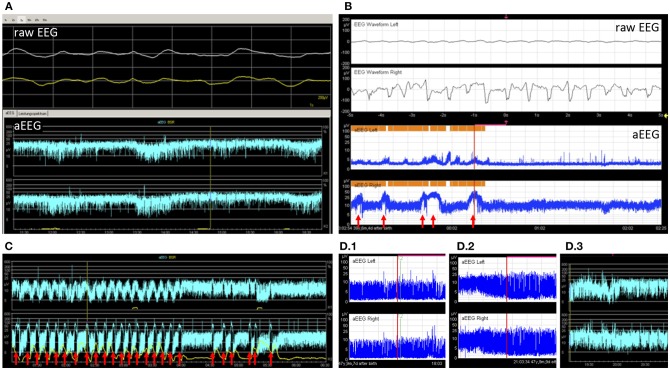
Example tracings. **(A)** 2 9/12 years old girl after resuscitation and bifrontal craniotomy. Despite very slow simultaneous waves in the raw EEG, the aEEG tracing showed continuous normal voltage. Without the raw EEG this pattern could have been mistaken for a physiological pattern (patient 26). **(B)** A 5 months old toddler with large left-sided intracranial hemorrhage. The aEEG showed side difference with continuous pattern and no seizures on the healthy side and flat trace and epileptic state on the affected side (patient 8). Seizures are marked with arrows. **(C)** An 8-year-old boy with febrile infection-related epilepsy syndrome and refractory epileptic state. Response to treatment can be seen as a decrease in seizure frequency and eventually a pause (patient 20). Seizures are marked with arrows. **(D)** A 2 months old toddler showed burst suppression pattern and coma after intoxication with lorazepam (D.1). Over days, background pattern improved to discontinuous (D.2) and finally to continuous background pattern (D.3), while the patient became conscious again (patient 22).

Twenty five EEGs were performed within 24 h of aEEG recording. EEGs performed at a greater interval were not considered for this study. aEEG background pattern was altered in 15 recordings (43%), whereas 21 (84%) conventional EEGs were altered. Of these, 13 (62%) were correctly identified as altered by aEEG. Eight cases (38%) of pathological EEG were missed by aEEG. Of 4 (16%) normal EEGs, all were recognized as normal by aEEG. Sensitivity for recognition of altered EEG pattern by aEEG in these patients was only 62%, but specificity was 100%. Examples for background patterns are provided in Figures 3A,D.

#### Evolution of Background Pattern

Three children had only one section available and were not eligible for this analysis. Out of the remaining 24 children, 13 (54%) had normal background patterns at the beginning and end of tracing, while 2 (8%) showed normalization (example given in Figure 3D). 2 (8%) children showed persistent mild abnormality, 3 (13%) progressed to severe, and 1 (4%) improved without normalization. 2 (8%) children showed persistently severe background patterns. One child with unilateral intracranial hemorrhage showed persistently normal background pattern on the healthy side and persistent severely depressed background pattern on the injured side (Figure 3B).

#### Seizures

Clinical seizures were documented by the nurse or doctor in charge during 15 (43%) recordings. Electrographic seizures in the aEEG were found in 16 (46%) recordings. There were three recordings with single clinical seizures and two recordings with repetitive clinical seizures undetected in the corresponding aEEGs. 6 (17%) aEEG recordings showed electrographic seizures without clinical correlate of seizure.

10 (29%) children showed electrographic epileptic state during their aEEG recording; 4 (40%) of these also showed clinical epileptic state (see Table 2). The Kappa coefficient was 0.3702, indicating poor agreement between aEEG results and clinical evaluation. This is caused by the fact that aEEG enabled the diagnosis of epileptic state more frequently than clinical observation. Examples for seizures are provided in Figures 3B,C.

**Table 2:** Electrographic and clinical seizures.

	**Electrographic epileptic state**	**Repetitive electrographic seizures**	**Single electrographic seizure**	**No electrographic seizure**
No clinical seizure	5	1	0	14
Single clinical seizure	1	2	1	3
Repetitive clinical seizures	0	2	0	2
Clinical epileptic state	4	0	0	0
Total (*n*)	10	5	1	19

#### Side Differences

Only 2 children showed side differences in their aEEG recordings, although 5 (14%) aEEGs were performed in patients with a unilateral process proven by cranial imaging (MRI/CT scan). Both children with side differences in aEEG had unilateral processes (see Figure 3B for an example).

### Therapeutic Consequences

We frequently initiated or adjusted antiepileptic treatment after detecting seizures by aEEG. aEEG findings were confirmed by EEG.

### Survival, Clinical Parameters, and aEEG Results

Survival of the included children was significantly associated with male gender (*p* = 0.0473), pathological background pattern in aEEG (*p* = 0.0402), and evolution of background pattern in aEEG (*p* = 0.0213). The following clinical parameters or aEEGs result were not significantly associated with survival: neurologic symptoms upon admission (*p* = 0.6280), sedation (*p* = 0.5660), mechanical ventilation (*p* = 1.0000), cranial imaging (CT/MRI scan; *p* = 1.0000) and unilateral process shown in imaging (*p* = 1.0000), seizures in aEEG (*p* = 0.2262), clinical seizures (*p* = 0.8890), pathological EEG (*p* = 0.3918), and side difference in at least one aEEG section (*p* = 1.0000).

## Discussion

So far, reports about aEEG in pediatric intensive care are scarce and include case reports about therapy guidance in seizures, after cardiac arrest and a study about neurodevelopmental outcome after cardiac surgery (20, 21, 26). In our cohort of critically ill children we show that aEEG is a valuable tool for continuous cerebral function monitoring. It facilitates identification of (subclinical) seizures and non-convulsive epileptic state. Depressed background activity is negatively associated with survival. The spectrum of patients treated in our PICU includes children with hepathological, oncological, neurosurgical, nephrological, and neurological conditions. Patients included in this study represent the heterogeneous collective of a tertiary center PICU with high rates of sedated patients, limiting the evaluation of electrocortical activity. Nonetheless, to our knowledge this study is unique, as it underlines the benefit of aEEG application in pediatric intensive care.

The number of included patients is low regarding the long study period. However, we found an increase in use over the years, suggesting that the benefits of continuous electroencephalographic monitoring have become more evident to our PICU team. A reason inhibiting even further use may be the circumstance that reference values for aEEG beyond 3.5 months of age have not been established (27). Reference values would greatly facilitate interpretation of aEEGs of children beyond the first few months of life. Due to the lack of established aEEG classifications in the pediatric population, we used the well-known neonatal classification by Hellström-Westas to categorize our findings (28). Similar approaches using this classification have been used to assess adults' aEEGs after cardiac arrest (12–15, 29). Both in children and adults, the prognostic value of continuous EEG and aEEG, respectively, has been shown after cardiac arrest (12, 16, 29). These studies evaluate the recovery time of CNV after return of spontaneous circulation. Unfortunately, there is no information available on “normal” aEEG findings in each specific age group. In our study, abnormal aEEG background pattern paralleled abnormal conventional EEG with high specificity. Despite the small number of patients, we conclude that in case of altered aEEG background, abnormalities may be expected in conventional EEG and should be confirmed by this method. Normal background in the aEEG on the other hand does not exclude abnormal findings in conventional EEG. We also found pathological background patterns and adverse evolution of background pattern to be negatively associated with survival. The risk for death was increased in patients with abnormal aEEG background pattern that deteriorated or failed to improve. Thereby both, pathological background pattern and evolution of background pattern, represent a simple method to estimate outcome in pediatric intensive care patients. Sensitivity for the detection of abnormal background pattern by aEEG compared to conventional EEG was only 62% in our study. This might be improved after establishment of age-specific reference values.

Side differences occurred only in two out of five patients with confirmed unilateral intracranial lesions. In full-term neonates, side differences in aEEG and especially unilateral seizures are known to be associated with unilateral brain injury (30). In children, side differences in conventional EEG are also associated with structural lesions and require cerebral imaging if newly diagnosed. To our knowledge, information about the diagnostic value of side differences in pediatric amplitude-integrated EEG is not available. From the present data and our results, we conclude that unilateral brain injury should be suspected in case of side differences after exclusion of artifacts. This may help to set the indication for cerebral imaging (CT, MRI) in older children where cerebral ultrasound is no option. However, side differences in aEEG background pattern cannot be recommended as screening tool for unilateral brain injury.

Epileptic state can either be convulsive or non-convulsive. Of special interest in pediatric critical care is the identification of non-convulsive epileptic state, as this condition is associated with an increased risk of in-hospital mortality (17, 18). Time from PICU admission to initiation of continuous (conventional) EEG monitoring is associated with mortality in children with electrographic ES (18). A frequent cause of non-convulsive ES is a preceding convulsive ES, as well as structural lesions and central nervous system (CNS) infections (17, 31, 32). For these reasons, early recognition of non-convulsive epileptic state is inevitable to initiate treatment as soon as possible. In our study, 80% of aEEGs were conducted to monitor and manage seizures or epileptic state. Seizure detection was higher by aEEG than by clinical observation, increasing the number of identified epileptic states from 4 (clinical) to 10 (electrographic). This is in accordance with the work published by Du Pont-Thibodeau who reported good sensitivity for the recognition of seizures in aEEG after short-term training of staff (19). A similar study by Guan showed a high detection rate of epileptic state in pediatric patients after short term training (33). On the other hand, 5 clinical seizures were missed by aEEG in our study. This points out one of the major limitations of aEEG, as the small number of electrodes do not cover the entire brain surface. Therefore, using aEEG instead of continuous full channel EEG may cause seizures to remain unrecognized. aEEG is not meant to substitute EEG, but should be used supplementary to EEG. However, cEEG is a limited resource that is not broadly available. If choosing between no continuous EEG monitoring at all or continuous EEG with limited number of electrodes, aEEG may thus be a reasonable choice. Finally, it needs to be mentioned, that artifacts may be mistaken for seizures, if only the aEEG band is used to identify seizure activity. We recommend to assess of the raw EEG curve in case of suspected electrographic seizures. Conventional EEG needs to be performed as available to complete diagnostics.

aEEG use has several limitations: Qualitative interpretation is observer-dependent. Further limitations are added by the fact that interpretation of conventional EEG includes assessment of frequency and wave morphology rather than amplitude, qualities that are lost in aEEG due to time compression. The raw EEG curve can help to assess these characteristics, but requires knowledge about conventional EEG and provides less information than full-channel EEG. Furthermore, validated classifications for the assessment of aEEG tracings are only available for neonates and preterm infants.

The establishment of reference values would allow further scientific evaluation of amplitude-integrated EEG in older infants and children. As acquisition of aEEG data in healthy children is difficult (long-term), EEG recordings from clinical routine could be used to calculate the corresponding aEEG curve and help obtain normal values for the pediatric population. Yet aEEG interpretation in pediatric intensive care patients will remain challenging due to sedation, neurosurgical interventions requiring head dressings, head injury prohibiting standard placement of electrodes, intra- or extracranial hemorrhages influencing recorded amplitudes, or craniotomy.

## Conclusion

In conclusion, the use of aEEG in our PICU has increased in recent years, consistent with a growing body of evidence supporting cEEG monitoring in pediatric critical care. Amplitude-integrated EEG is useful for the confirmation of suspected seizures and the detection of non-convulsive seizures/epileptic state in pediatric intensive care patients. Consistent with this, seizure detection and therapy guidance are the main indications for aEEG monitoring in our PICU. The presence of abnormal background pattern and poor evolution of background pattern in children was negatively associated with survival in our cohort. From our data, we conclude that aEEG is a potential supplement to conventional EEG and an option for long-term cerebral function monitoring if continuous EEG is unavailable. Continued research in different PICU populations as well as the establishment of normal values for children are needed to determine the future role of aEEG in the pediatric population, especially the association between background pattern and outcome in critically ill children.

## Data Availability

The raw data supporting the conclusions of this manuscript will be made available by the authors, without undue reservation, to any qualified researcher.

## Ethics Statement

This study was carried out after approval by the local ethics committee (Ethikkommission der Universität Duisburg-Essen). Written informed consent was not obtained because the study was conducted retrospectively. Data were anonymized for analysis, as ordered by the ethics committee.

## Author Contributions

NB and HM collected clinical data and assessed aEEGs. IS-A assessed EEGs. CW performed statistical analyses. NB wrote the manuscript. HM, CD-S, ET, and UF-M contributed to interpret data and to prepare the manuscript.

### Conflict of Interest Statement

The authors declare that the research was conducted in the absence of any commercial or financial relationships that could be construed as a potential conflict of interest.
